# INVESTIGATING OLDER ADULTS’ PERSPECTIVES ON TELEHEALTH ROBOTICS

**DOI:** 10.1093/geroni/igac059.3016

**Published:** 2022-12-20

**Authors:** Samuel Olatunji, Husna Hussaini, Kenneth Blocker, Naveen Uppalapati, Girish Krishnan, Wendy Rogers

**Affiliations:** University of Illinois Urbana-Champaign, Champaign, Illinois, United States; University of Illinois Urbana-Champaign, Urbana Champaign, Illinois, United States; University of Illinois Urbana-Champaign, Champaign, Illinois, United States; University of Illinois at Urbana Champaign, Urbana Champaign, Illinois, United States; University of Illinois at Urbana Champaign, Urbana Champaign, Illinois, United States; University of Illinois Urbana Champaign, Champaign, Illinois, United States

## Abstract

The current social distancing directives have heightened the need for older adults to seek remote healthcare solutions. Telehealth robots could support home health by carrying out on-demand tasks for diagnosis and treatment at the user’s home through remote teleoperation by the healthcare provider. Acceptability of such telehealth robots by older adults is critical to their successful deployment. The overall goal of this research was to investigate older adults’ perceptions and attitudes towards a telehealth robot supporting health checkups at home. Specific objectives were to explore potential healthcare use cases for the robot; identify facilitators and barriers to its use; and elicit design requirements for use in healthcare contexts. We interviewed 5 men and 5 women (ages 66–73) after they viewed a series of videos demonstrating the potential uses of the telehealth robot. The older adults had positive first impressions towards the telehealth robot and were generally open to the idea of using it for telehealth tasks. They conveyed a high level of trust with the robot, especially if it were controlled by a healthcare provider, yet expressed concerns with privacy and security of health information that would need to be addressed in the design of security protocols. They described added value of the robot’s healthcare support while suggesting potential improvements to the robot. This research provided insights into older adults’ perceptions and attitudes towards a telehealth robot as well as identified potential healthcare use cases that would inform the design requirements for telehealth robots in different home healthcare contexts.

